# Autologous HER2 CMV bispecific CAR T cells are safe and demonstrate clinical benefit for glioblastoma in a Phase I trial.

**DOI:** 10.1186/2051-1426-3-S2-O11

**Published:** 2015-11-04

**Authors:** Nabil Ahmed, Vita Brawley, Meenakshi Hegde, Kevin Bielamowicz, Amanda Wakefield, Alexia Ghazi, Aidin Ashoori, Oumar Diouf, Claudia Gerken, Daniel Landi, Mamta Kalra, Zhongzhen Yi, Cliona Rooney, Gianpietro Dotti, Adrian Gee, Helen Heslop, Stephen Gottschalk, Suzanne Powell, Robert Grossman, Winfried Wels, Yzonne Kew, David Baskin, Jonathan Zhang, Pamela New, John Hicks

**Affiliations:** 1 Department of Pediatrics, Center for Cell and Gene Therapy, Baylor College of Medicine, Houston, TX, USA; 2 Baylor College of Medicine, Houston, TX, USA; 3 Baylor College of Medicine/Texas Children's Hospital, Houston, TX, USA; 4 Houston Methodist Hospital, Houston, TX, USA; 5 CGT Frankfurt, Frankfurt, Germany

## 

Glioblastoma (GBM) remains incurable with current standard-of-care therapies. Adoptive T cell transfer holds the promise to improve outcomes for GBM patients. We report on the results of the Phase I clinical study, NCT01109095, administering autologous CMV.pp65 T cells grafted with a second generation HER2 chimeric antigen receptor (CAR) with a CD28.zeta signaling domain to patients with progressive GBM.

Seventeen CMV-seropositive patients with radiologically progressive HER2+ GBM were enrolled. The median age was 49 years (range 11 to 71; 6 children; 11 adults). Children enrolled had significantly larger tumor volumes at infusion. A cell product was successfully generated for all patients from a peripheral blood draw (maximum 90mL). A median of 67% (range: 46-82) of T cells expressed the HER2 CAR, and exhibited a median 985.5 (range 390 to 1292) CMV.pp65 reactivity in an IFN-γ Elispot assay (SFC/10^5^ T cells). Infusions of 1x10^6^/m^2^-1x10^8^/m^2^ were well tolerated without severe adverse events or cytokine release syndrome. HER2 CMV T cells were detected in the peripheral blood for up to 12 weeks post infusion, as judged by rtPCR of a CAR-specific amplicon. Out of 16 evaluable patients, 8 had progressive disease, 8/16 patients had objective responses: 1 patient had a partial response with a ~62% reduction in tumor volume lasting 8 months, 7 patients had stable disease for more than 6 weeks (of these 5 were durable >10 weeks) and 3 subjects are currently with a follow up 24 to >30 months, after T cell infusion. The median survival was 11.6 months from infusion and 24.8 months from diagnosis. The median survival for adults was 30 months from diagnosis.

We conclude that systemically administered HER2 CAR CMV bispecific T cells are safe. A durable clinical benefit was observed in ~38% of patients.

## Trial Registration

ClinicalTrials.gov Identifier NCT01109095

